# Adverse obstetric outcomes in pregnancies resulting from oocyte donation: a retrospective cohort case study in Sweden

**DOI:** 10.1186/s12884-015-0687-9

**Published:** 2015-10-08

**Authors:** Evangelia Elenis, Agneta Skoog Svanberg, Claudia Lampic, Alkistis Skalkidou, Helena Åkerud, Gunilla Sydsjö

**Affiliations:** Department of Women’s and Children’s Health, Uppsala University, Uppsala University Hospital, Uppsala, SE-751 83 Sweden; Department of Neurobiology, Care sciences and Society, Karolinska Institutet, Solna, Sweden; Obstetrics and Gynaecology, Department of Clinical Experimental Medicine, Faculty of Health Sciences, Linköping University, Linköping, Sweden

**Keywords:** Hypertensive disorders, Indication for oocyte donation, Oocyte donation, Pregnancy complications

## Abstract

**Background:**

Oocyte donation has been associated to gestational diabetes, hypertensive disorders, placental abnormalities, preterm delivery and increased rate of caesarean delivery while simultaneously being characterized by high rates of primiparity, advanced maternal age and multiple gestation constituting the individual risk of mode of conception difficult to assess. This study aims to explore obstetrical outcomes among relatively young women with optimal health status conceiving singletons with donated versus autologous oocytes (via IVF and spontaneously).

**Methods:**

National retrospective cohort case study involving 76 women conceiving with donated oocytes, 150 nulliparous women without infertility conceiving spontaneously and 63 women conceiving after non-donor IVF. Data on obstetric outcomes were retrieved from the National Birth Medical Register and the medical records of oocyte recipients from the treating University Hospitals of Sweden. Demographic and logistic regression analysis were performed to examine the association of mode of conception and obstetric outcomes.

**Results:**

Women conceiving with donated oocytes (OD) had a higher risk of hypertensive disorders [adjusted Odds Ratio (aOR) 2.84, 95 % CI (1.04–7.81)], oligohydramnios [aOR 12.74, 95 % CI (1.24–130.49)], postpartum hemorrhage [aOR 7.11, 95 % CI (2.02–24.97)] and retained placenta [aOR 6.71, 95 % CI (1.58–28.40)] when compared to women who conceived spontaneously, after adjusting for relevant covariates. Similar trends, though not statistically significant, were noted when comparing OD pregnant women to women who had undergone non-donor IVF. Caesarean delivery [aOR 2.95, 95 % CI (1.52–5.71); aOR 5.20, 95 % CI (2.21–12.22)] and induction of labor [aOR 3.00, 95 % CI (1.39–6.44); aOR 2.80, 95 % CI (1.10–7.08)] occurred more frequently in the OD group, compared to the group conceiving spontaneously and through IVF respectively. No differences in gestational length were noted between the groups. With regard to the indication of OD treatment, higher intervention was observed in women with diminished ovarian reserve but the risk for hypertensive disorders did not differ after adjustment.

**Conclusion:**

The selection process of recipients for medically indicated oocyte donation treatment in Sweden seems to be effective in excluding women with severe comorbidities. Nevertheless, oocyte recipients-despite being relatively young and of optimal health status- need careful counseling preconceptionally and closer monitoring prenatally for the development of hypertensive disorders.

## Background

Oocyte donation (OD) is a well-established form of infertility treatment for women with premature ovarian failure, which may be caused by idiopathic or iatrogenic (after chemotherapy/ radiation/ surgery) diminished ovarian reserve, Turner syndrome, repeated unsuccessful IVF treatments and inheritable genetic maternal disorders [1]. In some countries, women with natural menopause can also receive treatment with donated oocytes.

Since the introduction of oocyte donation, there have been conflicting reports about the possible overrepresentation of this group among those presenting with complications during pregnancy and delivery. Several studies have suggested that an increase in the incidence of gestational diabetes [2], hypertensive disorders [3–8], placental abnormalities [9], preterm delivery [9] and increased rate of caesarean delivery [9] may be related to this treatment. According to research on immunological aspects of OD pregnancies, some clinical complications may theoretically arise, as the embryo resulting from oocyte donation is immunologically unrelated to the mother and this difference might predispose to placental pathology [10, 11]. On the other side, pregnancies following oocyte donation are often characterized by high rates of primiparity and multiple gestation [6, 12], factors that might introduce a bias when assessing the association between OD and pregnancy complications.

Sweden first permitted oocyte donation in 2003, solely on the basis of medical indication; oocyte donation now represents approximately 2.5 % of the total IVF and ICSI treatment cycles [13] with a total pregnancy and live birth rate per embryo transfer at about 30 %. Reproductive centers in Sweden, i.e. the University clinics that are allowed to perform IVF treatment with donated oocytes, practice mostly single embryo transfer (SET) and therefore the multiple pregnancy rate in IVF settings overall is now 4.8 % of all pregnancies [13]. It should be stressed that in 2010, only 10 % of oocyte donation cycles in Sweden were performed in women older than 40 years, which constitutes by far the lowest rate in Europe [14, 15].

To date, no previous studies have compared the association between oocyte donation and obstetric outcomes in a national setting. Thus the aim of this study was to investigate if singleton pregnancies following oocyte donation based on medical indication in a sample of Swedish women with optimal health status in fertile age are more often associated with adverse obstetric outcomes compared to (i) naturally conceived pregnancies (in nulliparous women without infertility) and (ii) pregnancies conceived after non donor IVF. Furthermore, we aimed to study whether outcomes differed depending on the specific indication leading to treatment.

## Methods

### Study sample and data collection

The present study is part of the *“Swedish multicenter study on gamete donation”,* a cohort study of donors and recipients of donated gametes receiving treatment at fertility clinics performing donation treatment in Sweden, at the University Hospitals in Stockholm, Gothenburg, Uppsala, Umeå, Linköping, Örebro and Malmö. Subfertile couples are accepted for inclusion on the gamete donation program after medical and psychological assessment performed at the treating clinics. During the period 2005–2008, consecutive couples starting donation treatment were approached regarding participation. The Index group comprises of women who later gave birth to one child following treatment with donated oocytes. Women who did not speak and/or read Swedish were excluded [16]. Written and oral information was given and participants signed an informed consent form allowing the research group to have access to the medical records.

Two control groups were used in order to assess the outcome;

- Nulliparous women (Control group A) with spontaneously conceived pregnancies, singleton deliveries and no history of subfertility found in the medical register. All controls in group A were matched to the Index group in regard to age in three categories, ≤29, 30–34, ≥35 years, at a ratio of 2:1. With the exception of the eligibility criteria according to study design, Control group A was otherwise selected randomly. Unidentifiable information on the study subjects of Control group A was obtained and thus personal informed consent was not necessary for that group.
- Heterosexual women (Control group B) undergoing in vitro fertilization (IVF) treatment with their own gametes due to couple infertility at the University hospitals mentioned above. All Swedish speaking women receiving traditional IVF treatment concurrently to the Index group were approached regarding participation on the “*Swedish multicentre study on gamete donation”* and constituted the original control cohort [16]. However solely those who conceived with singleton pregnancies during and on the imminent study period were finally included in Control group B. Age matching was not performed. The women were given written and oral information about the study and informed consent was obtained.

All medical data analyzed were retrieved from the Swedish Medical Birth Register (MBR), a Swedish population-based register started in 1973 and held by the Swedish National Board of Health and Welfare. MBR, which is a validated register, includes information beginning with prenatal care and continuing through the delivery care and neonatal care [17–19]. Other medical information such as the treatment indication for the oocyte recipients originates from their treatment protocol after scrutinization of the medical record at each center.

The rationale for also including heterosexual women undergoing IVF as a control group was in order to investigate if the increased risks for oocyte recipients reported previously are attributable solely to donation. IVF pregnancies with autologous gametes are nowadays considered to be hampered by the underlying infertility, the characteristics of the infertile couple and/or the use of assisted reproductive techniques (i.e. conventional IVF or ICSI technique, fresh or frozen-thawed embryos) [20].

Our series include no maternal deaths. However one fetal intrauterine death in Control group A occurred on the 29^th^ week of gestation.

### Outcome measures

The medical data studied were based on the diagnosis according to the tenth version of the International Classification of Diseases (ICD-10) that the woman had received on the MBR. The following variables referring to medical practices and complications were studied: Mode of delivery [subdivided into Non Emergency and Emergency Caesarean Section, Normal Vaginal Delivery, Instrumental Delivery (with Vacuum extraction)], Induction of labor, Pregnancy Induced Hypertension, Preeclampsia, Eclampsia, HELLP syndrome or Hypertensive disorders of pregnancy as a whole (including all of the latter), Small for Gestational Age (SGA), Large for Gestational Age (LGA), Oligohydramnios, Polyhydramnios, Uterine Inertia, Fetal distress (either due to non reassuring heart beat on cardiotocography or acidemia/acidosis on fetal scalp blood test), Placenta praevia, Placental abruption, Retained Placenta with or without bleeding, Hemorrhage after labor, Nitrous Oxide gas or Epidural Anesthesia, Obstetrical lacerations of third or fourth grade, Total maternal hospital stay (from delivery date until hospital discharge). It should be noted that SGA and LGA were defined as a birth weight < −2SD or > +2 SD of the mean weight respectively as calculated by ultrasound scan compared to the expected value for the gestational length according to the Swedish growth standard [21]. Gestational age at delivery estimation was based on second-trimester ultrasound scan, or if this was not available then based on last menstrual period. For women who underwent in vitro fertilization and oocyte donation, gestational age was also calculated from the date of the embryo transfer. The ultrasound scan which is performed by specialized personnel during the 16^th^–19^th^ gestational week constitutes common practice in Sweden and is attended by 98 % of pregnant women [22].

The medical indications that led to oocyte donation were also studied. Poor responders were classified according to the Bologna criteria [23] i.e. women with high FSH levels menstrual cycle day 3–5, women with cancelled IVF treatment due to suboptimal response as well as those with idiopathic premature ovarian insufficiency. The category “egg factor” is generally poorly defined but it is widely associated to oocyte-related infertility with sufficient quantity of oocytes but somehow defective quality.

### Statistical analysis

All statistical analyses were performed using IBM SPSS v.20 (IBM Inc., Armonk, NY, USA). In all analyses, a *p*-value of <0.05 (two-sided) was considered statistically significant. Demographic and clinical characteristics were compared between Index women and Control group A and B respectively, using Student’s *t*-test (normally distributed variables) and Mann-Whitney *U* test (non-normally distributed variables) for continuous data and Chi square test for categorical data. Afterwards the association between various obstetric outcomes and index/control group status was studied by first comparing oocyte recipients to spontaneously pregnant women (Control group A) and then oocyte recipients to women having conceived with conventional non-donor IVF (Control group B). The comparisons were performed with the use of Chi square or Fisher’s exact test as well as logistic regression analyses; first a single regression model, i.e. without adjustment for socio-demographic or birth characteristics and afterwards by composing a multiple logistic regression model. A number of possible covariates based on results from previous studies were considered for inclusion in the multiple logistic regression model: maternal age as completed years on delivery day (two categories, <35 years or ≥35 years); body mass index (BMI, kg/m^2^) defined as BMI recorded at first antenatal visit (two categories, <25 kg/m^2^ or ≥ 25 kg/m^2^); nicotine use as either smoking cigarettes or smokeless tobacco (“*snus”*) (no/yes) defined as smoker 3 months before conception or at first visit to the prenatal center in gestational week 10; gestational length (continuous variable); and presence of chronic medical conditions (no/yes). For the purpose of comparison, women who conceived spontaneously (Control group A) or by conventional IVF (Control group B) were referred to as having an odds ratio of 1.0. The odds ratios and corresponding 95 % confidence intervals were calculated.

### Sensitivity analysis

According to the literature, nulliparous women have a higher risk for all adverse outcomes (i.e. nulliparity triples the risk for preeclampsia [24]). As our study group consisted of 92.1 % nulliparous (in contrast to 100 % in Control group A) we performed a sensitivity analysis in order to assess its possible influence. Parity did not appear to influence the obstetric outcomes. Thus, we did not adjust for parity and we intentionally included multiparous women in the analysis, even though the risk for adverse perinatal outcomes is lower, in order not to compromise sample size, while minimizing any risks for false associations.

## Details of ethics approval

The study was approved by the Regional Ethical Review Board in Linköping, Sweden (Nr M29–05, T113–07 and Nr 2012/289–32).

## Results

Demographic data and obstetric characteristics concerning oocyte recipients (Index group), Control group A (spontaneous conception) and Control group B (non donor IVF) are summarized in Table 1. Although the median age differed between Index women and Control Group A, no significant differences were noted after stratification (initial matching according to study design). Regarding parity, all 150 women (100 %) in Control group A were nulliparous, in contrast to 58/63 women (92.1 %) in Control Group B and 70/76 women (92.1 %) of the Index group (5 multiparous with parity 2–3 and 6 multiparous with parity 2–4 respectively).

**Table 1:** Demographic and obstetrical data for oocyte recipients (Index group) and Control groups A and B

	Index group (*n* = 76)		Control group A (*n* = 150)		Control group B (*n* = 63)	
	Median (IQR)	Min-Max	Median (IQR)	Min-Max	Median (IQR)	Min-Max
Age, years	35.0 (4.0)	25–43	34.0 (4.0)***	19–36	33.0 (5.0)*	25–39
<35	36(47.4 %)		82(54.7 %)		42(66.7 %)*	
≥35	40(52.6 %)		68(45.3 %)		21(33.3 %)*	
	Median (IQR)	Min-Max	Median (IQR)	Min-Max	Median (IQR)	Min-Max
Estimated gestational age, week	40.0 (4.0)	28–42	40.0 (3.0)	28–43	39.0 (2.0)	36–42
	Mean (SD)	Min-Max	Mean (SD)	Min-Max	Mean (SD)	Min-Max
BMI, kg/m^2^	24.98(3.65)	18.0–34.6	24.32 (3.93)	18.1–41.0	24.06(3.54)	16.0–32.6
<25	33(47.1 %)		87(65.9 %)*		40(64.5 %)*	
≥25	37(52.9 %)		45(34.1 %)*		22(35.5 %)*	
Nulliparity	70/76(92.1 %)		150/150 (100 %)		58/63(92.1 %)	
Nicotine use						
No	63/68(92.6 %)		110/141(78 %)**		56/61(91.8 %)	
Yes	5 /68(7.4 %)		31/141 (22 %)**		5/61 (8.2 %)	
Chronic diseases						
No	55/76(72.4 %)		131/150(87.3 %)*		54/63(85.7 %)	
Yes	21/76(27.6 %)		19/150 (12.7 %)*		9/63 (14.3 %)	

*p < 0.05

**p < 0.01

***p < 0.001

The chronic medical conditions reported were asthma, pre-gestational diabetes mellitus, hypothyroidism, epilepsy, mental health disorders, Crohn’s disease, Systemic Lupus Erythematosus (SLE), Inflammatory Systemic Disease, Rheumatoid Arthritis (RA), renal disease (renal agenesis, renal insufficiency and renal transplantation), anemia, thrombosis and hematological diseases (data not shown). It should be noted that the prevalence of these conditions did not differ between the groups with the exception of hypothyroidism (7 oocyte recipients *vs* 1 in Control Group A and 0 in Control Group B), 40 % of which could be attributed to women with Turner syndrome, possibly due to careful pre-pregnancy screening of Turner women in Sweden [25]. University clinics are almost unanimous that women with severe comorbidities should be declined treatment; thus, only relatively healthy women were included in the Index group.

Tables 2 and 3 describe obstetric outcomes, either medical practices or complications, for the Index group and Control groups A and B. Women who underwent oocyte donation had overall more pregnancy complications compared to both Control groups (Tables 2 and 3). In particular, oligohydramnios was diagnosed more often among Index group versus Control group A (9.2 % *vs* 0.7 % respectively) [adjusted OR = 12.74, 95 % CI (1.24–130.49)]. The overrepresentation of oligohydramnios among the Index population might in part reflect an overdiagnosis due to frequent ultrasound scans on this group. Cesarean section was performed more often among women who conceived with donated oocytes (55.3 %) *vs* pregnant women with autologous oocytes (26 % in Control group A and 19 % in Control group B). It should be noted that when compared to Control group A, oocyte recipients had a higher risk for non emergency Cesarean section [aOR 5.13, 95 % CI(2.00–13.17)], whereas when compared to Control group B had higher risk for emergency Cesarean Section [aOR 15.98, 95 % CI (3.27–78.23)]. The prevalence of hypertensive disorders of pregnancy in women after oocyte donation (Index group) was 2.5-fold higher than in patients with spontaneous conception (Control group A) (15.8 % versus 6 %; *p* = 0.017). There were no differences in prevalence of eclampsia and pregnancy-induced hypertension. The preeclampsia rates were 13.2 % for oocyte recipients, whereas only 6 % for women having conceived spontaneously and 9.5 % for IVF women (Tables 2 and 3) which are comparable to that of the general obstetric population in Sweden (2–10 %) [26, 27]. It should be observed that when gestational hypertensive disorders were analysed independently and not as a unified group, statistical significance was not reached possibly due to the relatively small number of cases in each category. Nevertheless, no associations were found regarding complications such as pre-gestational and gestational diabetes mellitus, placenta praevia and placental abruption, SGA or LGA infant, polyhydramnios and obstetrical lacerations of 3^rd^ or 4^th^ grade (data not shown). Furthermore medical practices such as use of epidural analgesia or nitrous oxide use during active labor did not differ between groups.

**Table 2:** Unadjusted and adjusted odds ratios (OR) and corresponding 95 % confidence intervals (CI) for delivery related outcomes for women treated with donated oocytes (Index group) compared to women with no history of infertility (Control group A)

Outcome	Index group n/N (%)	Control group A, n/N (%)	Crude OR (95 % CI)	Adjusted OR (95 % CI)
Normal delivery	21/76 (27.6 %)***	90/150 (60 %)	0.26 (0.14–0.50)*	0.30 (0.15–0.60)**
Instrumental delivery^a^	13/58 (22.4 %)	20/142 (14.1 %)	1.77 (0.74–4.25)	1.95 (0.75–5.09)
Caesarean Section (CS)	42/76 (55.3 %)***	39/150 (26 %)	3.69 (1.97–6.92)***	2.95 (1.52–5.71)**
Non emergency CS	18/76 (23.7 %)***	8/150 (5.3 %)	5.79 (2.36–14.22)***	5.13 (2.00–13.17)**
Emergency CS^a^	24/58(41.4 %)**	31/142(21.8 %)	2.49(1.20–5.13)**	1.93(0.88–4.22)
Induction of labor^a^	33/58 (56.9 %)***	31/142 (21.8 %)	3.34 (1.63–6.82)***	3.00 (1.39–6.44)**
Uterine Inertia, primary-secondary^a^	13/58 (22.4 %)	32/142 (22.5 %)	0.82 (0.36–1.84)	0.88 (0.36–2.16)
Hypertensive disorders of pregnancy^f^	12/76 (15.8 %)*	9/150 (6 %)	3.42 (1.32–8.86)*	2.84 (1.04–7.81)*
Pregnancy-Induced Hypertension	2/76 (2.6 %)	0/150	–	–
Preeclampsia	10/76 (13.2 %)	9/150 (6 %)	2.37 (0.92–6.12)	2.41 (0.84–6.89)
Fetal distress^a^	16/58 (27.6 %)*	21/142 (14.8 %)	2.62 (1.11–6.19)*	1.96 (0.78–4.97)
Very Preterm birth (<32 weeks)	3/76 (3.9 %)	3/150 (2.0 %)	6.19 (0.63–60.76)	6.48 (0.61–68.56)^b^
Preterm birth (<37 weeks)	13/76 (17.1 %)	13/150 (8.7 %)	2.42 (0.97–6.05)	1.86 (0.70–4.95)^b^
Maternal Hospitalization post partum				
*≥*2 days	73/75(97.3 %)**	127/148 (85.8 %)	10.40 (1.36–79.80)*	4.47 (0.53–37.76)^c^
*≥*3 days	59/75(78.7 %)**	86/148 (58.1 %)	2.64 (1.33–5.26)**	1.14 (0.51–2.58)^c^
Post-partum hemorrhage	12/76 (15.8 %)**	7/150 (4.7 %)	3.29 (1.12–9.70)**	7.11(2.02–24.97)^d^**
Retained placenta^e^	8/34 (23.5 %)**	4/111 (3.6 %)	7.58 (2.03–28.30)**	6.71 (1.58–28.40)*

All outcomes are adjusted for maternal age (<35, ≥35 years), BMI (<25 or ≥25 kg/m^2^), Nicotine Use (Yes/No), Gestational length (continuous variable, weeks), Chronic diseases (Yes/No)

^a^Excluding all non-emergency Caesarean Section (CS)

^b^Not adjusted for gestational length but for all other covariates

^c^Adjusted for usual covariates, CS and Hypertensive disorders

^d^Adjusted for usual covariates and CS

^e^Excluding all CS

^f^Hypertensive disorders including preeclampsia, pregnancy induced hypertension, eclampsia or HELLP

*p < 0.05

**p < 0.01

***p < 0.001

**Table 3:** Unadjusted and adjusted Odds Ratios (OR) and corresponding 95 % confidence intervals (CI) for delivery related outcomes for women treated with donated oocytes (Index group) compared to women treated with traditional IVF (Control group B)

Outcome	Index group n/N(%)	Control group B, n/N(%)	Crude OR (95 % CI)	Adjusted OR (95 % CI)
Normal delivery	21/76(27.6 %)***	39/63(61.9 %)	0.25 (0.12–0.53)***	0.29 (0.13–0.65)**
Instrumental delivery^a^	13/58 (22.4 %)	12/53(22.6 %)	0.90(0.35–2.33)	0.86 (0.29–2.55)
Caesarean Section (CS)	42/76(55.3 %)***	12/63 (19 %)	6.01 (2.65–13.59)***	5.20 (2.21–12.22)***
Non emergency CS	18/76 (23.7 %)	10/63(15.9 %)	2.21 (0.91–5.40)	1.82 (0.71–4.66)
Emergency CS^a^	24/58(41.4 %)***	2/53 (3.8 %)	16.96 (3.68–78.24)***	15.98 (3.27–78.23)**
Induction of labor^a^	33/58(56.9 %)***	12/53(22.6 %)	3.19 (1.35–7.56)***	2.80 (1.10–7.08)*
Uterine Inertia, primary-secondary^a^	13/58 (22.4 %)*	4/53 (7.5 %)	3.24 (0.94–11.17)	3.67 (0.92–14.66)
Hypertensive disorders of pregnancy^f^	12/76 (15.8 %)	6/63 (9.5 %)	2.08 (0.73–5.93)	1.66 (0.54–5.08)
Pregnancy-Induced Hypertension	2/76 (2.6 %)	0	–	–
Preeclampsia	10/76 (13.2 %)	6/63 (9.5 %)	1.44 (0.49–4.21)	1.39 (0.44–4.42)
Fetal distress^a^	16/58 (27.6 %)*	5/53 (9.4 %)	3.22 (1.04–9.99)*	2.86 (0.83–9.86)
Very preterm birth (<32 weeks)	3/76 (3.9 %)	0	–	–
Preterm birth (<37 weeks)	13/76 (17.1 %)*	3/63 (4.8 %)	3.94 (1.04–14.88)*	3.45 (0.88–13.62)^b^
Maternal Hospitalization post partum				
*≥*2 days	73/75 (97.3 %)*	54/63(85.7 %)	10.87 (1.34–90.91)*	5.26 (0.55–50.35)^c^
*≥*3 days	59/75(78.7 %)**	35/63(55.6 %)	3.03 (1.39–6.58)**	1.74 (0.70–4.34)^c^
Post-partum hemorrhage	12/76 (15.8 %)	6/63 (6.5 %)	1.47 (0.49–4.42)	3.67(1.03–13.03) ^d^*
Retained placenta^e^	8/34 (23.5 %)	4/51 (7.8 %)	3.83 (1.01–14.53)*	2.98 (0.73–12.18)

All outcomes are adjusted for maternal age (<35, ≥35 years), BMI (<25 or ≥25 kg/m^2^), Nicotine Use (Yes/No), Gestational length (continuous variable, weeks), Chronic diseases (Yes/No)

^a^Exclude all non-emergency Caesarean Section (CS)

^b^Non adjusted for gestational length but for all other covariates

^c^Adjusted for usual covariates, CS and Hypertensive disorders

^d^Adjusted for usual covariates and CS

^e^Exclude all CS

^f^Hypertensive disorders including preeclampsia, pregnancy induced hypertension,eclampsia or HELLP

*p < 0.05

**p < 0.01

***p < 0.001

In Table 4, the distribution of the medical indications that led to oocyte donation treatment among oocyte recipients is presented. The most common reason for receiving donated oocytes was premature ovarian failure or being “poor responder” [23] (48.7 %) followed by Turner syndrome (13.2 %) and bilateral oophorectomy or post chemotherapy (11.8 %).

**Table 4:** Indication to infertility treatment among participating women in the oocyte donation group

Indication to oocyte donation	n	Percent (%)
Poor responder & Premature Ovarian Insufficiency (POI)	37	48.7
Turner syndrome	10	13.2
After oophorectomy/chemotherapy	9	11.8
“Egg factor”	6	7.9
Multiple unsuccessful IVF cycles	5	6.6
Genetic reasons	5	6.6
Unclassified	4	5.3
Total	76	100.0

The category “egg factor refers to oocyte-related infertility with sufficient quantity of oocytes that were somehow defective in quality

In Table 5, a subgroup analysis within the Index group based on the indication of treatment and compared to Control group A is presented. The analysis revealed that induction of labor and caesarean section occurred more frequently among the group of women with premature ovarian insufficiency or who were “poor responders” (45.9 and 56.8 % respectively) compared to other indications of OD treatment (41.2 and 50 % respectively) or spontaneously conceived pregnancies (Table 5). Risk for postpartum hemorrhaging was highest for the subgroup “other indication” of OD treatment.

**Table 5:** Unadjusted and adjusted Odds Ratios (OR) and corresponding 95 % confidence intervals (CI) for delivery related complications for women treated with donated oocytes compared to women who conceived spontaneously (Control group A), reported by cause for oocyte donation

	Oocyte recipients other^a^		Oocyte recipients POI^b^	
	Crude OR (95 % CI)	Adjusted OR (95 % CI)	Crude OR (95 % CI)	Adjusted OR (95 % CI)
Hypertensive disorders	4.13(1.30–13.05)*	3.13 (0.94–10.48)	3.49(1.12–10.92)*	3.42 (0.99–11.87)
Induction of labour^c^	2.53 (0.93–6.93)**	2.59 (0.88–7.65)	4.11(1.66–10.22)**	3.33 (1.25–8.82)*
Cesarean section	2.79 (1.21–6.46)*	2.35 (0.98–5.62)	4.08 (1.82–9.15)**	3.03 (1.28–7.15)*
Post-partum hemorrhage	3.42 (0.90–13.03)	7.04 (1.45–34.16)*^d^	2.93 (0.78–11.07)	5.97(1.11–32.15)*^d^
Uterine inertia^c^	0.36 (0.08–1.64)	0.42 (0.09–2.07)	1.01 (0.37–2.78)	0.93 (0.30–2.84)

^a^Women with Turner syndrome, oophorectomy/chemotherapy, “egg factor”, multiple unsuccessful IVF cycles, genetic reasons, unclassified

^b^POI: Premature ovarian insufficiency and poor responders

All outcomes are adjusted for maternal age (<35, ≥35 years), BMI (<25 or ≥25 kg/m^2^), Nicotine Use (No/Yes), Gestational length (continuous variable, weeks), Chronic diseases (No/Yes)

^c^Exclude all non-emergency CS

^d^Model adjusted for CS also

*p < 0.05

**p < 0.01

***p < 0.001

## Discussion

Our analysis provides evidence that oocyte donation is associated with hypertensive disorders of pregnancy, oligohydramnios, induction of labor, delivery by caesarean section, retained placenta, post-partum hemorrhage and longer hospital stay after delivery even though oocyte recipients in our study are quite healthy and relatively young. The association between oocyte donation and hypertensive disorders of pregnancy remained significant even after adjustment for various covariates.

Although several previous studies investigating obstetric and perinatal pregnancy outcomes after oocyte donation have been performed, most of them lacked an appropriate control group; thus the only studies with a design similar to ours are the ones by Malchau et al. and Stoop et al. Malchau et al. [8] investigated perinatal outcomes in 375 pregnancies after oocyte donation in a Danish national cohort study and showed two- to threefold increased risk regarding hypertensive disorders and caesarean section in OD pregnancies when compared to IVF/ICSI and spontaneously conceived singleton pregnancies. It should however be noted that our study population, despite being of similar ethnical origin, comprises women younger than the women in the Danish cohort [8], thus strengthening the reported results. Stoop et al. [7], performed a matched-pair analysis with regard to age, ethnicity, parity and plurality between OD and IVF conceived women with autologous oocytes. They reported a similar trend towards a higher incidence of pregnancy-induced hypertension, preterm birth, caesarean section and instrumental delivery in the OD study group [7]. In contrast to some previous reports, we did not demonstrate any increased risk for gestational diabetes among women having conceived through oocyte donation, possibly due to the lower age and even perhaps lower weight of our population [28–30].

Caesarean section was performed more often among oocyte recipients than controls in a national setting where vaginal delivery (84 % of all singleton deliveries in Sweden) is the mode of delivery of first choice [31]. It should, however, be noted that the high proportion of cesarean section cannot be solely attributed to the obstetrician’s or woman’s choice since elective (humanitarian) caesarean section did not differ between the groups (data not shown). Furthermore, the period during which they stayed at the hospital after delivery (≥3 days) was longer in the study group but the risk was eliminated after adjusting for the presence of hypertensive disorders, caesarean section and the usual covariates. Trends were also noted regarding preterm birth and very preterm birth but the overall gestational length did not differ between the groups. High risk for post partum hemorrhage was observed even after adjusting for operative delivery with greater prevalence than expected by previous reports [25, 32].

Finally, after investigating adverse obstetric outcomes with regard to the indication of OD treatment, a tendency towards greater maternal complications was observed for women with declining ovarian reserve. This is partly in accordance with the findings of Keegan et al. [33] and Pados et al. [34] who demonstrated that young oocyte recipients exhibited the highest rates of gestational hypertension and preeclampsia, which indicates a possible relationship between diminished ovarian function and hypertensive disorders. However, caution is advised in the interpretation of the data due to the limited size of the study population, as seen by the wide confidence interval.

### Strengths and limitations of the study

The major strength of our study is its national design comprising all centers allowed to perform IVF treatment with donated oocytes in Sweden and hence recruiting a wide range of women from both urban and rural areas. Furthermore it is one of the few that compares three modes of conception [6, 8] and constitutes one of the largest series on singletons pregnancies after oocyte donation published thus far, with a well defined and age matched control group [7, 8] where the medical indication of the oocyte donation treatment for every woman was taken into account. In addition, in Sweden, antenatal care is standardized and free of charge with good availability of the public health system independently of social or employment status and educational level of the pregnant woman. Thus, the differences noted on the various outcomes cannot be vastly attributed to different level of obstetric care given. Finally, our study group comprises relatively young recipient women with ascertained health status with donated oocytes derived from young (≤35 years) fertile women and not through egg sharing from other infertile women undergoing ART. It should be stressed that the participating clinics at the nationwide oocyte donation program, despite not having a standardized way of making the evaluation of the recipients but rather following their own clinical policy, seem nevertheless to be unanimous regarding the importance of relatively young age and good health status of the oocyte recipients.

One of the limitations of our study is the lack of power, as shown by the wide confidence intervals, which may limit the interpretation of some results. Moreover, due to the retrospective design of our study and because infertility is often unreported in obstetric records, we cannot exclude the possibility that there might be women with a history of infertility in Control group A. On the other hand, this fact would only lead to underestimation of associations. Finally in the assessment of the various outcomes, we did not take into account parameters such as donor age, paternal age, ART method (conventional IVF or ICSI) as well as if the pregnancy resulted from a cryopreserved or fresh embryo. It should however be noted that large series did not find donor age to have a significant association with perinatal outcome [7, 35]; this according to the authors might in part reflect the homogeneity of the oocyte donor population i.e. more than 98 % of donors reported being younger than 35 years. Similar conditions apply even in Sweden where according to common practice in the various IVF university clinics the vast majority of donors are anonymous, younger than 35 years and with proven fertility. In particular, the nationwide study on oocyte donation in Sweden reported a mean age of 31.5 ± 4.6 years for oocyte donors supporting the conclusions deducted [36].

## Conclusion

Our study confirms that the evaluation process of oocyte recipients in Sweden appears to be successful in excluding women with severe comorbidities. However, oocyte recipients based on medical indication, despite being of young age and optimal health, have higher risks for adverse obstetric outcomes compared to women conceiving with autologous oocytes, regardless of mode of conception. Obstetricians should therefore provide careful counseling preconceptionally and closer monitoring prenatally regarding the development of hypertensive disorders.

## Abbreviations

ART: Assisted reproductive technology
CS: Caesarean section
IVF: In vitro fertilization
LGA: Large for Gestational Age
MBR: Medical Birth Register
OD: Oocyte Donation
SGA: Small for Gestational Age
